# Mechanotransduction at focal adhesions: integrating cytoskeletal mechanics in migrating cells

**DOI:** 10.1111/jcmm.12054

**Published:** 2013-04-04

**Authors:** Jean-Cheng Kuo

**Affiliations:** Institute of Biochemistry and Molecular Biology, National Yang-Ming UniversityTaipei, Taiwan

**Keywords:** cell migration, focal adhesions, actin cytoskeleton

## Abstract

Focal adhesions (FAs) are complex plasma membrane-associated macromolecular assemblies that serve to physically connect the actin cytoskeleton to integrins that engage with the surrounding extracellular matrix (ECM). FAs undergo maturation wherein they grow and change composition differentially to provide traction and to transduce the signals that drive cell migration, which is crucial to various biological processes, including development, wound healing and cancer metastasis. FA-related signalling networks dynamically modulate the strength of the linkage between integrin and actin and control the organization of the actin cytoskeleton. In this review, we have summarized a number of recent investigations exploring how FA composition is affected by the mechanical forces that transduce signalling networks to modulate cellular function and drive cell migration. Understanding the fundamental mechanisms of how force governs adhesion signalling provides insights that will allow the manipulation of cell migration and help to control migration-related human diseases.

- Introduction
- Focal adhesions are regulated by mechanical forces
- Focal adhesions-transduced signals regulate cytoskeletal mechanics
- Signals targeting focal adhesions drive cell migration
- Conclusion and future prospects

## Introduction

Cell migration is a fundamental phenomenon that controls multiple biological processes, including embryonic development (morphogenesis), wound healing and immune responses 1. During development, dividing cells migrate to mediate various processes ranging from gastrulation to organogenesis. In addition, when there is injury to the skin or another tissue, cells migrate there to repair the damage. These include platelets, which migrate and aggregate at the injury site to stop bleeding by forming fibrin clots, macrophages and neutrophils, which migrate to kill microorganisms that cause infection, and fibroblasts and epithelial cells, which migrate to the damaged structures and provide cover for the creation of new tissue. These cells can migrate to their destinations individually over long distances or as epithelial sheets, and on arrival they perform specific functions. In both situations, the cell migration cycle is similar and is controlled by complex pathways 1, 2.

The cell migration cycle consists of the extension of the leading edge, formation of new adhesions, translocation of the cell body and detachment of the trailing edge of the cell. To achieve all the steps of the cycle, the actin cytoskeleton and adhesion organelles are reorganized spatio-temporally. When cell migration begins, dendritic actin networks are assembled by polymerizing actin filaments at the leading edge to push the membrane forward 3. This significant force involved in pushing a cell's leading edge does not involve myosin II motors acting on the actin cytoskeleton 4–7. Soon after the membrane at the leading edge protrudes, adhesion organelles are formed to attach the protrusion to the substratum. Subsequently, the actomyosin contractile force is generated by myosin II motors sliding on actin filaments, which promotes bundling of filamentous actin (stress fibres) that connect distal points of adhesions; this allows the contractile forces to propagate across the cell, and applies the force to the substratum through the adhesions; the result is that the cell body is pulled forward 8. Finally, the disassembly of adhesions at the trailing edge leads to detachment of the cell at the rear. Therefore, the dynamic response of the actin cytoskeleton and adhesion organelles is fundamental to coordinating the entire process of cell migration.

The adhesion organelles that allow cells to adhere to the substratum, which also mediate the signals that regulate cell migration, are the integrin-based FAs. FAs form when the central component, the integrin receptor, is activated by engagement with the ECM onto the substratum, which then recruits numerous FA-associated proteins to connect with the actin cytoskeleton 9–11. At the last count 12, 180 proteins had been reported to be associated with FAs to make up the integrin adhesome 12, 13; these include cytoskeletal proteins, adaptor proteins, and signalling proteins, such as kinases, phosphatases, phospholipases and regulators of small guanosine triphosphatase (GTPases). This complex molecular ensemble produces the signalling that regulates the dynamics of FAs, controls the integrity of the linkage between integrin and actin and organizes of the actin cytoskeleton; these together coordinately control cell migration 9–11, 14–18. Cell migration is central in many biological processes and disease states, and therefore an understanding of what is known about the regulation of FAs provides a resource that should help to control abnormal migration.

## Focal adhesions are regulated by mechanical forces

**Fig. 1:**
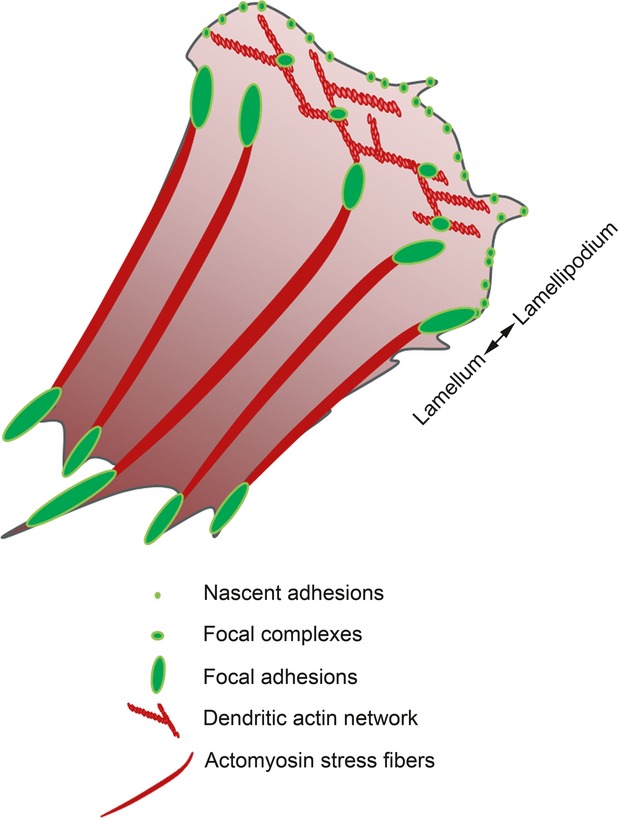
Schematic representation of the structures of the actin cytoskeleton and focal adhesions (FAs). The maturation of FAs is differentially coupled to the specific organization of actin cytoskeleton.

The signalling networks in FAs are modulated by a process called FA maturation 19. During maturation, FAs grow in size and change composition after which they either stabilize or begin to disassemble. Based on their size (∼0.1–10 μm^2^) and localization, FAs can be classified into nascent adhesions, focal complexes and FAs (Fig. 1). Nascent adhesions assemble soon after the integrin receptors engage with the ECM at the edge of lamellipodium, and are either undergoing fast turnover during active protrusions or are evolving into focal complexes within the lamellipodial dendritic actin network. At the lamellipodium-lamellum interface, these adhesions grow and elongate into FAs that are connected by bundles of actin filaments (stress fibres), which serve to anchor the cell 4, 20, 21. All classes of FAs depend on maturation stimuli for their formation and maintenance.

The maturation stimuli can be supplied through biochemical or physical cues. Biochemical regulators of FA maturation include small G-proteins of the Rho-family, which transduce signals to regulate assembly and dynamics of FAs 20, 21. Previous studies have shown that the formation of focal complexes is signalled by the activity of small GTPase Rac1 22, while RhoA signalling promotes the formation of long-lived FAs through activating myosin II-driven contractility 23, 24. GTP-bound RhoA activates its target, Rho-associated kinase (ROCK); this increases myosin II-mediated contractility by inhibiting the myosin light chain phosphatase and directly phosphorylating myosin II regulatory light chain (MLC) 25, 26. The myosin II-generated contractile force along actin filaments provides the major cellular tension that drives FA maturation 19. Physical cues include the cellular tension generated directly from actomyosin contractility, which is also altered by ECM rigidity through feedback loops to modulate the pulling forces exerted by the cells 27–31, and forces from outside of the cell, such as variation in shear forces. Therefore, FAs are really individual mechanosensors whose maturation state is indicative of the local balance with respect to the mechanical forces generated from cellular tension or from external forces 32.

Focal adhesions in different maturation states are composed of specific protein components, which are determined by the local mechanical force 33, 34. However, it is unlikely that all proteins directly sense the mechanical force; rather the recruitment of proteins into FAs is a hierarchical cascade driven by a number of force-sensitive FA proteins 35–38. In response to mechanical force, these force-sensitive FA proteins may undergo structural rearrangement or enzymatic modification that change their binding preferences with respect to other FA-associated proteins (force-responsive FA proteins) and this then further modulates the protein association with FAs. The abundance of these proteins in FAs mainly acts to strengthen the linkage between integrin and actin filaments 39–41.

The proteins that could serve as force-sensitive or force-responsive FA proteins consist of subsets of scaffolding and regulatory proteins. The scaffolding proteins are able to physically connect the actin cytoskeleton to integrin receptors *via* direct or indirect interactions, while the regulatory proteins control the connection between integrin receptors and actin filaments through their abilities to modulate the activity, stability or functionality of the components in the scaffolding group. The scaffolding proteins include actin-binding proteins and adaptors. Specifically, the actin-binding proteins include proteins that are able to bind directly to the cytoplasmic domains of integrin receptors, such as talin 41–43, α-actinin 41, 44–46, and filamin A/B/C 41, 47–50, or that are able to connect with integrin receptors *via* other actin-binding proteins or adaptors, such as vinculin 39, 41, VASP 41, 51, 52 and zyxin 41, 52, 53. The adaptors are FA proteins containing specific domains, including src homology 2 (SH2), src homology 3 (SH3), pleckstrin homology (PH), LIM, FERM and calponin homology (CH) domains. The SH2 domain typically binds a phosphorylated tyrosine residue present on its target protein 54, 55, while the classic SH3 domain uses proline-rich peptides as its binding partners 56. PH domains can bind phosphatidylinositol lipid within biological membranes, such as phosphatidylinositol (3,4,5)-trisphosphate and phosphatidylinositol (4,5)-bisphosphate; it thus plays a role in recruiting proteins to specific membranes sites 57, 58. LIM domains have highly divergent sequences that are composed of two contiguous zinc finger motifs with a two-amino acid residue hydrophobic linker 59; these function as a protein-binding interface within many subcellular components such as FAs 60. Evidence indicates that some LIM domain-containing proteins are highly dependent on myosin II activity for FA abundance, suggesting that these proteins may undergo force-dependent unfolding to unmask the binding sites that mediate mechanotransduction 33, 34, 38. FERM domains consist of three modules (the F1, F2 and F3 subdomains) that are able to form a clover-shape structure 61; they play an important role in certain FA proteins that are able to recognize the cytoplasmic tail of β-integrin and mediate integrin activation, such as talin 42, 43 and kindlin 62–65. CH domains are mainly involved in actin binding 66. Altogether, the FA proteins in the scaffolding group may involve force-triggered unfolding or recruitment that promotes FA association of other components; these are able to produce a physical strengthening of the connection between the integrin receptors and actin filaments.

The regulatory proteins are FA components that modulate FA integrity *via* their enzymatic activity; they include the proteins with small GTPase activity, guanine nucleotide exchange factor (GEF) activity, GTPase-activating protein (GAP) activity, proteolytic activity and activity that regulate protein phosphorylation states. The GTPase activity of the Rho-family proteins, which includes Rac1 and RhoA, is critical for FA maturation and actin cytoskeleton organization 22, 23, 67. The activity of these GTPases is known to be regulated *via* a switchable cycle that involves GEFs that exchange bound GDP for GTP for activation, and GAPs that promote intrinsic GTP hydrolysis for inactivation 68, 69. Thus, the abundance of GEFs and GAPs regulates the organization of FAs and the actin cytoskeleton through a modulation of GTPase activity. The proteins with proteolytic activity function by cleaving the proteins within FAs, thereby disrupting the linkage between integrin and actin, which allows disassembly of FAs. For example, the Ca^2+^-dependent cysteine-type protease calpain mediates FA disassembly 70–72 *via* irreversibly cleaving several FA scaffolding proteins, including integrin 73, 74, paxillin 70 and talin 70, 75. In addition, the proteolytic activity of calpain also regulates the activities of protein tyrosine kinases, such as FAK (focal adhesion kinase) 70, 76, 77 and SRC 78, as well as protein tyrosine phosphatases, such as PTP-1B 78. The activities of various kinases (tyrosine kinases and serine/threonine kinases) and phosphatases (tyrosine phosphatases and serine/threonine phosphatases) trigger signalling cascades 79, 80 that control FA dynamics 81, 82.

**Fig. 2:**
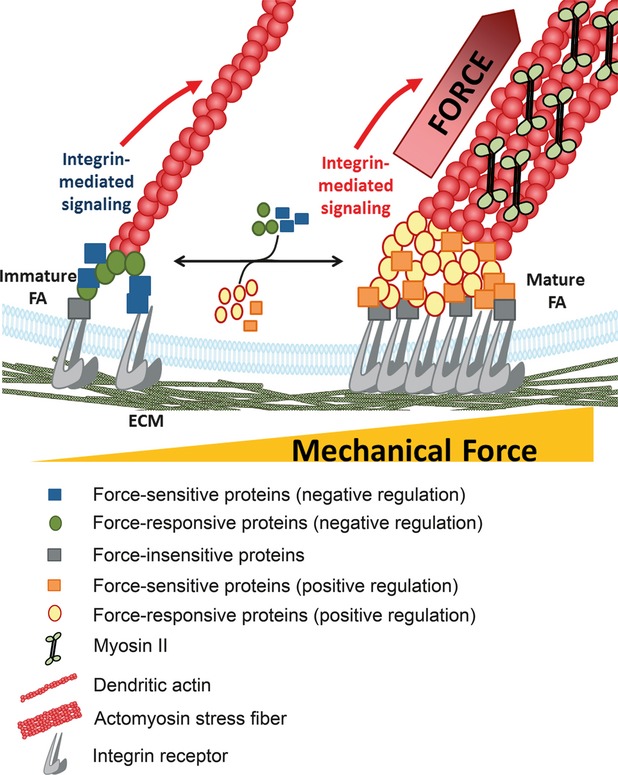
Schematic representation of how the protein composition of FAs is re-organized in response to mechanical force. Focal adhesion protein composition is altered by mechanical force. Within immature FAs, force-insensitive proteins (grey squares), force-sensitive proteins (blue shapes) and force-responsive proteins (green shapes) coordinately transmit the specific integrin-mediated signals. In response to mechanical force, focal adhesion abundance of force-sensitive proteins (blue shapes) and force-responsive proteins (green shapes) are decreased, while the abundance of force-sensitive proteins (orange shapes) and force-responsive proteins (yellow shapes) are increased. The proteins have similar levels of abundance between immature and mature FAs that are considered as force-insensitive proteins (grey squares).

Understanding the mechanical force-induced compositional changes in FAs provides information on the molecular complexity, diversity and signals of the integrin-mediated adhesions. The proteins that show increased force-dependent FA abundance could be either positively or negatively regulated by force; these include force-sensitive or force-responsive FA proteins (Fig. 2). To date, many studies based on microscopy and proteomics have revealed that changes in FA components occur in response to mechanical force. To understand how FA-related signalling networks modulate the strength of the linkage between integrin and actin, the force-dependent FA abundance of scaffolding and regulatory proteins is organized, as shown in Table 1. This provides a broad view of our understanding of how FAs enable cells to respond to their mechanical environment *via* modulation of their composition in a hierarchical cascade.

**Table 1:** Force-dependent focal adhesions abundance of scaffolding and regulatory proteins. The lists of scaffolding and regulatory proteins are classified into two classes: FA abundance positively regulated by force and FA abundance negatively regulated by force. The proteins in each class could contain force-sensitive or force-responsive proteins

Scaffolding protein	
FA abundance positively regulated by force	ABLIM 34, ACTN1 16, 33, 34, ACTN4 33, 34, CNN1 33, 34, CNN2 33, 34, CNN3 33, 34, CORO1C 33, 34, CSRP1 33, 34, CSRP2 33, 34, FBLIM1 33, 34, FHL2 33, 34, FHL3 33, 34, FLNA 33, 34, 114, FLNB 33, 34, FLNC 33, 34, DAB2 33, 34, LIMA1 33, 34, LIMCH1 33, LIMD1 34, LMO7 33, LPP 33, 34, MYH9 33, 34, NCK1 34, PDLIM1 33, 34, PDLIM2 34, PDLIM4 33, 34, PDLIM5 33, 34, PDLIM7 33, 34, PLEC1 33, 34, 115, SH3BP4 33, SORBS3 33, 34, SPTAN1 33, TES 17, 33, 34, 116, TGFB1I1 33, 34, TLN1 33, 34, TRIP6 33, 34, VCL 33, 34, 39, ZYX 16, 17, 33, 34, 92
FA abundance negatively regulated by force	ARP2/3 complex 33, 117, CAPZB 33, CRIP2 33, DBNL 33, EPB41 33, EPS8 33, 34, FHL1 33, MICALL1 33, TNS3 33
Regulatory protein	
FA abundance positively regulated by force	ARF1 33, ARF6 33, 34, CAPN1 33, CAPN2 33, CAPN5 33, CSK 34, DDR2 33, GIT1 33, GIT2 33, 34, GNA11 33, GNA12 33, GNA13 33, GNAQ 33, GNB1 33, GNB2 33, 34, ILK 33, 34, JAK1 33, PDGFRB 33, PTK2 34, PTPN11 34, PTPN2 34, PTPN12 34, RAB1B 33, 34, RAB14 33, 34, RAB18 33, 34, RAB21 33, 34, RAB23 33, 34, RAB3B 33, RAB34 33, 34, RAB35 33, 34, RALA 33, 34, RALB 33, 34, RAP1B 33, RAP2B 33, RHOA 33, RHOB 33, ROR2 33, RRAS2 33, 34, SRC 34, YES1 33
FA abundance negatively regulated by force	ARHGEF7 33, CSNK2A1 33, KRAS 33, 34, NRAS 33, PPP2CB 33, PTPRF 33, PTP4A2 33, PTPRK 33, RAB11B 33, 34, RAB13 33, RAB8A 33, RAN 33, TENC1 33

## Focal adhesions-transduced signals regulate cytoskeletal mechanics

Focal adhesion components comprise the linkage between integrin receptors and the actin cytoskeleton and these dictate FAs dynamics (the formation, maturation and disassembly of FAs) as well as cytoskeletal organization. The initial linkage between integrin and actin is built *via* a FA adaptor, talin, which activates integrin receptor by binding to its cytoplasmic domain (NPXY motif) and also connects to actin filaments 42, 43. Myosin II-mediated contractile force reinforces the linkage by modulating FA composition *via* a hierarchical cascade. For example, force-dependent talin unfolding reinforces the linkage by binding to the actin-binding protein, vinculin 39. In addition, myosin II-dependent recruitment of the actin-binding proteins, filamin-A/B/C and the adaptor, migfilin, strengthens the linkage between integrin and actin filaments *via* a connection that links the integrin receptors indirectly *via* a FA adaptor, kindlin-2 83, 84.

Mechanical force modulates the integrin-mediated signals transduced from the force-sensitive and force-responsive FA proteins. In response to myosin II activity, the abundance of RhoA enhancers, such as TRIP6 (thyroid hormone receptor interactor 6) 85, testin 86 and GEF-H1 87, is increased in FAs. In addition, FA abundance of actin-bundling proteins, such as α-actinin 88, synaptopodin-2 89 and supervillin 90, 91 as well as several cytoskeletal LIM domain-containing adaptors 33, 34, 38, such as zyxin 92–94, PDLIM1 95, PDLIM2 95, PDLIM4 95, PDLIM5 95, PDLIM7 95 and FHL2 96, is enhanced. This suggests that mechanical force could promote the level of cellular tension in a positive feedback loop through promoting the association of specific FA components that allows the maturation of FAs and creates bundles of filamentous actin (stress fibres) 33.

Cellular tension also contributes to FA turnover 97, as mature FAs disassembly is blocked by myosin II inhibition 98. Previous experiments have revealed that the Ca^2+^-activated protease calpain mediates proteolysis of FA proteins 71, 72, 75 and endocytosis-mediated pathways are able to recycle FA components; these serve as important mediators in regulating the disassembly of FAs 99, 100. Some disassembly factors are recruited to mature FAs 33, which may explain how actomyosin contractility mediates FA turnover at the retracting edge of the cells.

Myosin II-mediated contractile force also influences the protein association of immature FAs that transduce signals to promote lamellipodial protrusion 33, 101. In the lamellipodium, actin is arranged as a dendritic network by continuous actin polymerization 3. This cytoskeletal structure is mainly regulated by the Rho GTPase Rac1, but is also induced by myosin II inhibition 5. Inhibition of actomyosin contractility enhances the abundance into immature FAs of Rac1 activators, such as RacGEF β-PIX (PAK-interacting exchange factor-β) 102, RacGEF modulator EPS8 (epidermal growth factor receptor pathway substrate 8) 103, MIF (macrophage migration inhibitory factor) 104 and PKA (protein kinase A) 105, of Rac1 downstream effectors, such as IRSp53 (insulin receptor tyrosine kinase substrate p53) 106 and N-WASP (neuronal Wiskott–Aldrich Syndrome protein) 106, 107, and of Rac1 downstream targets, such as Arp2/3 complex 108, cofilin 109 and the actin monomer binding protein Cap1 110. Previous studies have shown that the Arp2/3 complex serves as the primary mediator of actin polymerization during lamellipodial protrusion, and Rac1 is sufficient to induce Arp2/3-dependent lamellipodium extension *via* the Rac1 downstream effectors, IRSP53 and N-WASP. FA association of the actin depolymerization factor cofilin promotes actin polymerization at the lamellipodia through the generation of new barbed ends for binding and this affects the Arp2/3 complex. Therefore, FA association of the Rac1 regulatory modules within the immature FAs explains the negative feedback mechanism of actomyosin contractility on the propagation of continuous membrane protrusions 33. Taken together, the biochemical signals associated with FAs are adjusted by the local balance of mechanical forces; this dictates FA dynamics, cytoskeletal organization and the nature of cellular tension.

## Signals targeting focal adhesions drive cell migration

Cell migration, a highly dynamic and well regulated process, consists of well-defined steps that include the following: extension of the leading edge and the formation of immature FAs; FA maturation and cell body translocation; the FA disassembly and rear retraction. Integrin-mediated signals from the FAs steps (assembly, maturation and disassembly), which are adjusted by the local balance of cellular tension and the mechanical properties of the environment, regulate actin polymerization and organization. During the migrating cycle, FA dynamics and cytoskeletal organization conjoin to drive this coordinated process 111.

The initial step of the migration cycle is the extension of the leading edge and formation of nascent adhesions (immature FAs) beneath the lamellipodium. These nascent adhesions not only stabilize the protrusion, but also transduce specific signals that continuously promote membrane protrusion. The protein components of nascent adhesions include the Rac1 regulatory module (Rac1 activators, Rac1 downstream effectors and Rac1 downstream targets), which promotes dendritic/branched actin polymerization for continuous protrusion extension, and positively enhances the assembly of immature FAs (nascent adhesions and focal complexes) 33, 101. Soon after, the immature FAs connect with bundles of actin filaments at the lamellipodia-lamella interface and they undergo a compositional reorganization and enlarge into mature FAs. This compositional reorganization includes force-sensitive and force-responsive FA proteins; these coordinate to reinforce the linkage between integrin and actin, help to form mature FAs and aid bundling of filamentous actin (stress fibres) 33, 34, 38.

The RhoA regulatory module associated with mature FAs activates myosin II through the action of downstream effector, ROCK, on up-regulating of MLC phosphorylation 24. Myosin II activation sustains the myosin II-mediated contractile force and this further enhances the magnitude of the cellular tension. This enhanced cellular tension transmits the pulling force along the actin bundles to the adhesion sites, thereby translocating the cell body forward. The last step of the migration cycle is disassembly of mature FAs at the cell rear, which is also contractile force-dependent 98. Actomyosin contractility promotes FA association with the disassembly factors, including proteases 71, 73, 75 and the components of endocytosis pathways 99, 100. This disrupts the linkage between integrin and actin by cleaving and recycling the structural proteins that form the mature FAs 33. Following the action of the disassembly factors, the pulling force supplied by the actomyosin contractility retracts the trailing edge of the cell, completing the migration cycle. Altogether, FAs not only serve as mechanosensors that re-organize their composition in response to mechanical forces, but also function as mechanotransducers that mediate specific cellular signalling pathways that regulate FA turnover and cytoskeletal organization, thereby controlling cell behaviour and driving cell migration.

## Conclusion and future prospects

In response to mechanical force, FAs reorganize their protein composition in a hierarchical cascade to assemble FAs in different maturation states. The proposed model is shown in Figure 2. Within immature and mature FAs, some FA-associated proteins have similar levels of abundance, indicating that they serve as force-insensitive proteins. Some FA-associated proteins (force-sensitive proteins) show negative or positive regulation in response to mechanical force, which may alter their FA abundance, conformation, or enzymatic activity, thereby changing the association of FAs with other FA proteins (force-responsive proteins) to assemble FAs in different maturation states. In immature FAs, force-insensitive proteins, force-sensitive proteins and force-responsive proteins coordinately transmit specific integrin-mediated signals to promote dendritic actin polymerization and the formation of immature FAs for membrane protrusion. In response to mechanical force, force-sensitive proteins in immature FAs are negatively regulated and decrease their FA abundance, thereby driving the dissociation of force-responsive proteins from FAs. By contrast, subjecting force-sensitive proteins in mature FAs to mechanical force enhances their FA abundance and triggers the association of force-responsive proteins to assemble mature FAs. FAs serve as force transmission pathways to sense the local balance of mechanical forces.

Focal adhesions enable cells to respond to their various environments, which contain diverse mechanical properties. They do this by manipulating their protein compositions, which allows the transmission of specific biochemical signals that mediate cellular behaviour. Within a range of tissue microenvironments, cells feel and sense the proper matrix elasticity, thus displaying their specific biological function in specific tissues. However, the mechanical properties of the matrix in some disordered tissues can mislead the cells and cause disease progression. For example, matrix remodelling and stiffening promote breast tumorigenesis and malignancy 112. In liver fibrosis, fibril-forming collagens facilitate further progression of chronic liver disease 113. Therefore, understanding the molecular details of how FAs respond to mechanical force will provide a resource that will aid the discovery of new therapeutic strategies. Although the details of the control of cellular phenomena *in vivo* are complicated, systems analysis using proteomics-related techniques, protein microarrays, or phospho-kinase antibody arrays is able to globally explore signalling modules and networks of FAs in the specific cells cultured under conditions of tissue-level matrix stiffness. For a particular signalling network, tracking a FA protein tagged with a fluorescent protein using microscopy-based technologies, such as live-cell imaging techniques, enables observation and quantification at high spatial and temporal resolution. Further illustration of the integrin-mediated signalling pathways in different cell types or under different physiological conditions will provide a possible foundation for designing therapeutic strategies for some human diseases.
